# Synthetic Knee MRI T_1p_ Maps as an Avenue for Clinical Translation of Quantitative Osteoarthritis Biomarkers

**DOI:** 10.3390/bioengineering11010017

**Published:** 2023-12-24

**Authors:** Michelle W. Tong, Aniket A. Tolpadi, Rupsa Bhattacharjee, Misung Han, Sharmila Majumdar, Valentina Pedoia

**Affiliations:** 1 Department of Radiology and Biomedical Imaging, University of California San Francisco, San Francisco, CA 94143, USAsharmila.majumdar@ucsf.edu (S.M.); valentina.pedoia@ucsf.edu (V.P.); 2 Department of Bioengineering, University of California Berkeley, Berkeley, CA 94720, USA

**Keywords:** T_1p_ map, T_2_ map, knee, MRI, osteoarthritis, synthesis, generative AI, deep learning, CNN, U-Net

## Abstract

A 2D U-Net was trained to generate synthetic T_1p_ maps from T_2_ maps for knee MRI to explore the feasibility of domain adaptation for enriching existing datasets and enabling rapid, reliable image reconstruction. The network was developed using 509 healthy contralateral and injured ipsilateral knee images from patients with ACL injuries and reconstruction surgeries acquired across three institutions. Network generalizability was evaluated on 343 knees acquired in a clinical setting and 46 knees from simultaneous bilateral acquisition in a research setting. The deep neural network synthesized high-fidelity reconstructions of T_1p_ maps, preserving textures and local T_1p_ elevation patterns in cartilage with a normalized mean square error of 2.4% and Pearson’s correlation coefficient of 0.93. Analysis of reconstructed T_1p_ maps within cartilage compartments revealed minimal bias (−0.10 ms), tight limits of agreement, and quantification error (5.7%) below the threshold for clinically significant change (6.42%) associated with osteoarthritis. In an out-of-distribution external test set, synthetic maps preserved T_1p_ textures, but exhibited increased bias and wider limits of agreement. This study demonstrates the capability of image synthesis to reduce acquisition time, derive meaningful information from existing datasets, and suggest a pathway for standardizing T_1p_ as a quantitative biomarker for osteoarthritis.

## 1. Introduction

Knee osteoarthritis (OA) is a leading cause of chronic disability and pain worldwide, impacting 23% of individuals over age 40 and reducing mobility for 80% of those diagnosed [1,2]. This irreversible condition is characterized by the degeneration of articular cartilage which significantly impacts quality of life and thus necessitates early detection. As individuals age and OA progresses, the structural integrity of the cartilage extracellular matrix declines, resulting in a reduced ability to retain water, proteoglycan disorganization, and cartilage degeneration [3,4]. Several risk factors that contribute to OA include age, weight (obesity), sex (female), prior knee injury, participation in high-impact sports, and occupations that involve heavy physical labor [5,6,7]. Intervention efforts aim to reduce OA progression through non-invasive and non-pharmacological interventions such as self-management, exercise, and weight loss. In cases of advanced OA, treatment strategies include non-steroid anti-inflammatory pain medication, intra-articular injections, and surgeries such as total knee arthroplasty [8,9,10].

Standard clinical diagnosis of OA involves evaluation of the patient’s age, self-reported symptoms, and morphological changes such as radiographic identification of osteophytes and joint-space narrowing [11,12]. However, these criteria are more characteristic of advanced disease once considerable damage has occurred and often require more invasive treatment strategies [12,13]. Furthermore, radiographic changes are poor predictors of cartilage loss [14] and have weak associations with symptoms [15,16]. MRI enables visualization of soft tissue in joint structures without radiation and achieves higher sensitivity to pathological changes indicative of OA [17,18]. However, the characterization of cartilage injury from structural MRI varies, leading to accurate assessments, instances of underestimation, and instances of overestimation across pathology and cartilage compartments [19,20,21].

Unlike radiography and structural MRI, compositional non-contrast MRI techniques such as T_1p_ and T_2_ mapping are sensitive to early biochemical changes in cartilage that precede morphological changes in OA [22,23,24,25]. T_2_ relaxation measures free water (65–80% cartilage total weight [3]) proton movement, and thus elevated values may indicate collagen/extracellular matrix degeneration [26]. T_1p_ relaxation describes spin-lattice relaxation in the rotating frame which captures changes between water protons and their macromolecular environment, mainly proteoglycans (10–15% cartilage wet weight [3]). Increases in T_1p_ values are associated with proteoglycan degeneration which is characteristic of OA, offering greater sensitivity to OA onset than T_2_ values. Additionally, T_1p_ values reflect patient-reported pain, symptoms, and loss of function (KOOS) [25,27,28].

T_1p_ mapping shows promise for early OA detection with slightly greater sensitivity than T_2_ [29], yet further validation of both sequences is required to increase confidence in T_1p_ maps as viable quantitative imaging biomarkers for clinical practice [30,31,32]. However, the addition of T_1p_ mapping to standard imaging protocols faces challenges, including prolonged image acquisition times, image processing requirements, and often SAR concerns that have prevented its widespread adoption in the clinic [33,34,35]. In contrast, T_2_ mapping has gained broader adoption for clinical and research purposes, often being acquired in the absence of T_1p_ mapping, as seen in large studies like the Osteoarthritis Initiative [36].

Image synthesis via deep learning has been gaining popularity in aiding clinical workflows by overcoming limitations with acquisition time, labor, and expenses [37,38]. In previous knee MRI synthesis studies, deep learning models were developed to generate images with new contrast [39], augment datasets by generating images with the same contrast [40], and standardize MR images to reduce scanner effects prior to downstream processing [41]. While newer architectures have emerged [42], convolutional neural networks (CNNs) remain valuable as a data-driven approach to learning image feature representations tailored to perform specific tasks well [43,44,45,46]. This study aims to generate synthetic T_1p_ maps from T_2_ maps to derive new information that can improve clinical outcomes and create possibilities for further analysis of large cohort studies.

This work makes the following contributions.

- This study proposes medical image synthesis as a repeatable and efficient method for extracting quantitative biomarkers. This methodology may overcome limitations in hardware acquisition speed, variations across scanner sites, and availability of quantitative imaging sequences in existing datasets or at scanner locations.
- To the best of the authors’ knowledge, this is the first study to synthesize T_1p_ maps from T_2_ maps for knee MRI scans. This contribution is valuable for characterizing and assessing T_1p_ as a biomarker for knee OA, as it reduces acquisition time and facilitates the extraction of meaningful information.
- While there is a substantial body of research on U-Nets for image segmentation, the utility of CNNs for image synthesis and clinical deployment is less known [45,46]. This study develops an image synthesis algorithm using well-studied network architecture and performs comprehensive evaluation across four diverse cohorts.
- This study provides clarity on the network’s ability to perform synthesis (1) within a well-constrained held-out test dataset and (2) in a new context where images were acquired under varied imaging conditions. These findings provide a greater understanding of the strengths and limitations of model architecture and the feasibility of clinical translation.

## 2. Materials and Methods

### 2.1. Cohort Description

After obtaining IRB approval for this retrospective study, 897 knee MRI scans were identified from four cohorts, spanning 594 healthy and diseased patients. For network development, 509 unilateral knees were used from two cohorts: (A) a UCSF study on ACL injury [47] and (B) a multi-center study conducted at UCSF (San Francisco, CA, USA), Mayo Clinic (Rochester, MN, USA), and Hospital for Special Surgery (New York, NY, USA) on recovery from ACL tears and reconstructive surgery [35,48]. Scans were acquired before, 6 months after, and 12 months after injury. Out-of-distribution performance was evaluated on external data from two cohorts: (C) 343 unilateral knee scans in a clinical setting and (D) bilateral knee scans acquired simultaneously from 23 subjects without ACL tear or reconstruction but with idiopathic OA at the knee or the hip. A description of the dataset is summarized in Table 1.

### 2.2. Image Acquisition and Processing

Scanner and coil array information for each cohort is specified in Table 1. All the scans were acquired at 3 Tesla. The network was trained with cohorts A and B, whose data was collected on three different scanners using a single type of knee coil. For cohort C, images were acquired as an add-on to the standard clinical procedure on one scanner using four different coils. For cohort D, images were acquired on a different scanner using two coil arrays simultaneously for bilateral knee image acquisition.

T_1p_/T_2_-weighted images were acquired from magnetization-prepared angle-modulated partitioned k-space spoiled gradient echo snapshots (MAPSS) in the sagittal plane [49]. For unilateral knee MRI (cohorts A–C), T_1p_ weighted MAPSS with fat suppression was performed at a spin-lock frequency of 500 Hz, and a spin lock time (TSL) over 0/2/4/8/12/20/40/80 or 0/10/40/80 ms. T_2_-weighted MAPSS was acquired at T2-preparation time (TE) of 0/12.9/25.7/51.4 ms or 2.9/13.6/24.3/45.6 ms. Other imaging parameters included time between magnetization preparations as TR = 1.2 s; FOV = 14 cm; slice thickness = 4 mm; acquisition matrix = 256 × 128; reconstructed matrix = 256 × 256; readout bandwidth = ±62.5 kHz; 5–10 ms TR (per view); 64 views per preparation; number of slices = 24; ky acceleration = 2). MAPSS was also used to acquire echo images for the bilateral knee scans (cohort D) with adjusted parameters: TSL = 0/10/40/80 ms only; TE = 0/12.9/25.7/51.4 ms only; increased slice number of 88; TR = 5.1 ms; 76 views per preparations; ky and kz accelerations of 2 × 3.

T_2_ and T_1p_ maps were calculated as follows: all relevant echo images were registered to the TE/TSL = 0 ms shared echo using 3D affine registration with a normalized mutual information criterion [50]. Prior to T_1p_ and T_2_ map generation, the bilateral scans were automatically divided into left and right unilateral scans. Levenberg–Marquardt mono-exponential fitting of registered echoes yielded ground truth T_1p_ and T_2_ maps [51].

### 2.3. Segmentation

Cartilage segmentations were obtained from the first echo (TE/TSL = 0 ms) using a 3D V-Net architecture trained on data from research studies. To achieve further granular analysis of cartilage compartments, an automatic segmentation algorithm [52] used the first echo (TE/TSL = 0 ms) images to identify cartilage compartment regions for the medial femoral condyle (MF), lateral femoral condyle (LF), medial tibial (MT), lateral tibial (LT), patellar (PAT), and trochlea (TRO) cartilage.

### 2.4. Training

**Figure 1:**
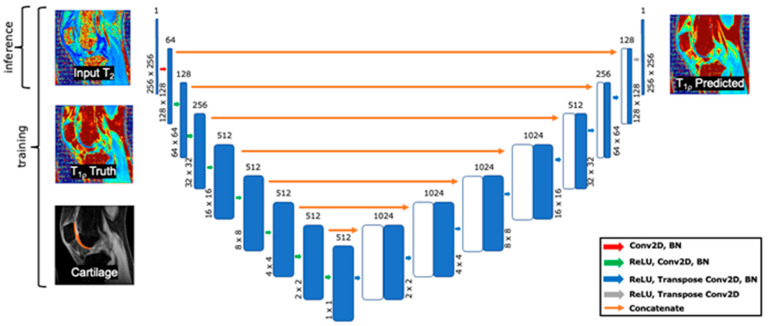
Synthetic T_1p_ maps were generated from T_2_ maps using this U-Net network. The optimal network that minimized the cartilage NMSE used combination of L1 and L2 loss in the cartilage and surrounding area.

Input T_2_ map slices from cohorts A and B were split into training, validation, and testing using a 65%:15%:20% split such that each subject was only in one subset and each study was similarly represented. T_1p_ map slices were predicted from T_2_ map slices using a 2D U-Net network (Figure 1) with 8 convolutional layers, ReLU activation, batch normalization, and skip connections. The network encodes T_2_ maps in a low-resolution high-dimensional space before upsampling the latent feature representations to predict T_1p_ maps. Network weights were optimized until validation loss stopped decreasing and 4-fold cross-validation was performed. A hyperparameter search identified the optimal loss function, input intensity scaling, and learning rate to minimize the normalized root mean squared error between the predicted and ground truth maps.

### 2.5. Within-Distribution Testing and Performance Evaluation for Image Generation

For model evaluation, the test dataset comprised 101 knees from 57 patients (cohorts A and B). Performance was evaluated across voxels in the entire imaging volume and within cartilage compartments using the NMSE, structural similarity (SSIM), peak signal-to-noise ratio (PSNR), Pearson’s correlation coefficient (CORR), and visual inspection. All models were implemented in Pytorch (Python 3.7; Pytorch 1.9.1, 1 GPU, 24 or 12 GB RAM).

The optimal network was selected based on the lowest NMSE in the cartilage segmentation and used a weighted loss function with L2 loss for the cartilage region and L1 loss for the remainder of the image Equation (1). Input T_2_ maps were clipped to values between 0 and 150 to reduce the effect of background noise.

(1) $$\mathrm{loss}=1.5\;\times \;L2\_\mathrm{loss}(y_{\mathrm{cartilage}},{\hat{y}}_{\mathrm{cartilage}})+L1\_\mathrm{loss}(y_{\mathrm{background}},{\hat{y}}_{\mathrm{background}}).$$

### 2.6. Out-of-Distribution Inference Testing for Model Generalizability

To evaluate model generalizability, inference was performed on data that differed from the training data. Whereas the model was trained on data collected using a single knee coil in a research environment (cohort A and B), synthetic T_1p_ maps were generated for data collected using various knee coils in a clinical setting (cohort C) and using two knee coils simultaneously in a research setting (cohort D) (Table 1).

### 2.7. Statistical Analysis: Quantitative Correlation

For both in-distribution and external cohort testing, Pearson’s correlation coefficients were calculated between the average synthesized and ground truth T_1p_ values in each cartilage compartment to assess the quality of synthesis [53]. Reported values include Pearson’s r to provide insight into the strength and direction of the relationship, the degrees of freedom that specify the dimensionality in which variance is estimated, and the two-tailed *p*-value to determine statistical significance (*p* < 0.001). Bland–Altman plots were generated for the average T_1p_ value in the cartilage compartments to demonstrate the spread as well as the limits of agreement [54,55]. The statistical testing was performed using Python (version 3.7).

## 3. Results

### 3.1. In-Distribution Cohort Test Set

#### 3.1.1. Example Demonstration

**Figure 2:**
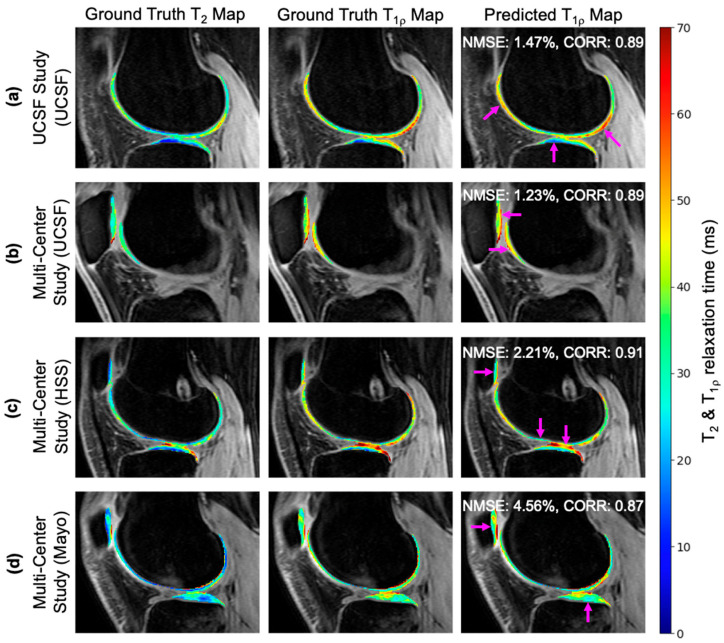
Four knees from patients who participated in one of two studies: (**a**) the UCSF (cohort A) study or (**b**–**d**) the multi-center (cohort B) study at one of three centers. Input ground truth T_2_ maps exhibit distinct intensity elevation and textural patterns compared to ground truth T_1p_ maps. Nevertheless, predicted T_1p_ maps generated by the CNN preserve these differences, as indicated by the regions marked by the arrows.

T_2_ input maps, ground truth T_1p_ maps, and predicted T_1p_ maps for four patients are shown in Figure 2. (a,c) Patients shown from a study at UCSF and HSS presented elevated T_1p_ values in the anterior and posterior cartilage relative to the central cartilage. (b,d) Patients shown from the multi-center study at UCSF and Mayo exhibited textural changes between the T_2_ and T_1p_ values in the patellar and trochlear cartilage. In all cases, the synthetic T_1p_ images maintained excellent reconstructions that captured the elevation patterns in ground truth images.

#### 3.1.2. Image Generation Performance Evaluation

Table 2 provides a summary of the NMSE, SSIM, PSNR, and CORR values across the entire test set and by cohort. The metrics are reported for the cartilage region and the entire imaging volume which includes muscle, bone, and background in addition to knee cartilage. Across all studies, the network performed well with low NMSE (2.41 ± 1.51%, range 2.18–2.61%) and strong correlation in the cartilage segmentation. Similarity metrics within each cartilage compartment (Table A1) found the PSNR of the UCSF study was 5.0 ± 2.0 higher than the multi-center study, with similar CORR values ranging from 0.81 to 0.9, and similar NMSEs ranging from 2.04% to 4.78% for all compartments except patellar cartilage from the multi-center study, which had a NMSE of 5.79%.

#### 3.1.3. Quantitative Correlation Analysis

**Figure 3:**
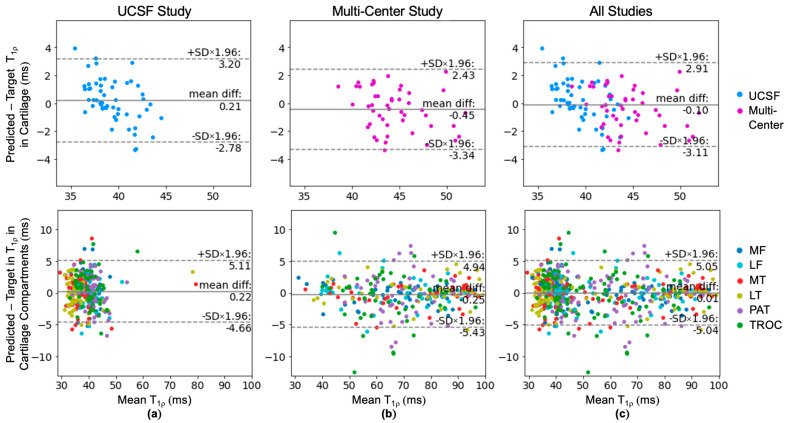
Bland-Altman plots for predicted T_1p_ performance across the entire cartilage tissue and within 6 cartilage compartments for (**a**) the UCSF study (cohort A), (**b**) the multi-center study (cohort B), and (**c**) all in-distribution studies (cohorts A and B). Model performance in each study reveals slight biases that were relatively consistent between the entire cartilage region and cartilage compartments. Across all studies, the network performed excellent synthesis with minimal bias and tight limits of agreement within a range that is clinically significant for cartilage region analysis.

**Figure 4:**
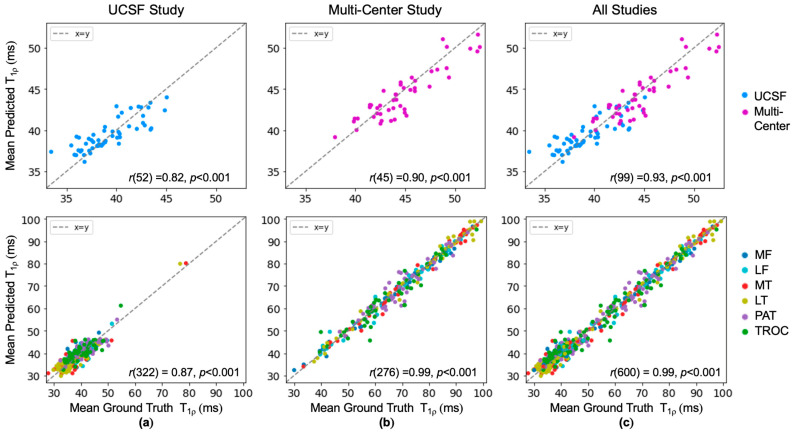
Ground truth T_1p_ and predicted T_1p_ values in knee cartilage were strongly correlated indicated by statistically significant Pearson’s r values for (**a**) the UCSF study (cohort A), (**b**) the multi-center study (cohort B), and (**c**) all in-distribution studies (cohorts A and B). Mean predicted T_1p_ values were close to the dashed unity line.

**Figure A1:**
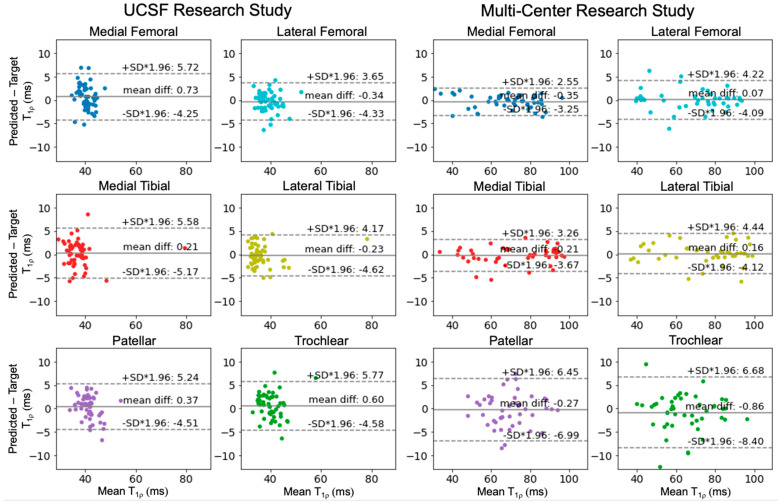
Bland-Altman plots for average T_1p_ relaxation times in six cartilage compartments indicated by color across two research studies (cohorts A and B). The solid line shows the bias and the dashed lines show the bias ± 1.96 times the standard deviation. Bias was minimal ranging from −0.86 to 0.73 ms well within the range of scan/re-scan reproducibility [58]. Limits of agreement were similar across the two studies (range ±2.90 to ±5.37 ms) despite varied MR scanner and were similar across compartments, apart from the patellar and trochlear cartilage having slightly wider limits (range ±4.87 to ±7.54 ms).

Bland–Altman plots of held-out test data in Figure 3 reveal minimal bias and tight limits of agreement across the entire cartilage region. Across each of the six cartilage compartments, bias remained minimal and limits of agreement were slightly wider than the entire cartilage region analysis. This discrepancy is likely attributed to fewer voxels in the compartment average. Data from the two in-distribution studies (cohorts A and B) have different ranges of ground truth T_1p_ values, with the mean T_1p_ for the multi-center study being 5.48 ms higher. Despite this difference, similar limits of agreement and biases indicate the network is robust to various values. The absence of underestimated low T_1p_ values suggests the network has learned a lower bound of relevant T_1p_ values, while higher T_1p_ values are well represented on both sides of the line of equality. Further examination of the cartilage compartments found the patellar and trochlear cartilage had wider limits of agreement than the other compartments. Within a study, mean T_1p_ values were similarly represented across all the cartilage compartments (Figure A1). Correlation plots show exceptional agreement across studies in all cartilage regions (Pearson’s r = 0.93) and cartilage compartments (Pearson’s r = 0.99) (Figure 4).

### 3.2. External Cohort Inference Set

#### 3.2.1. Example Demonstration

**Figure 5:**
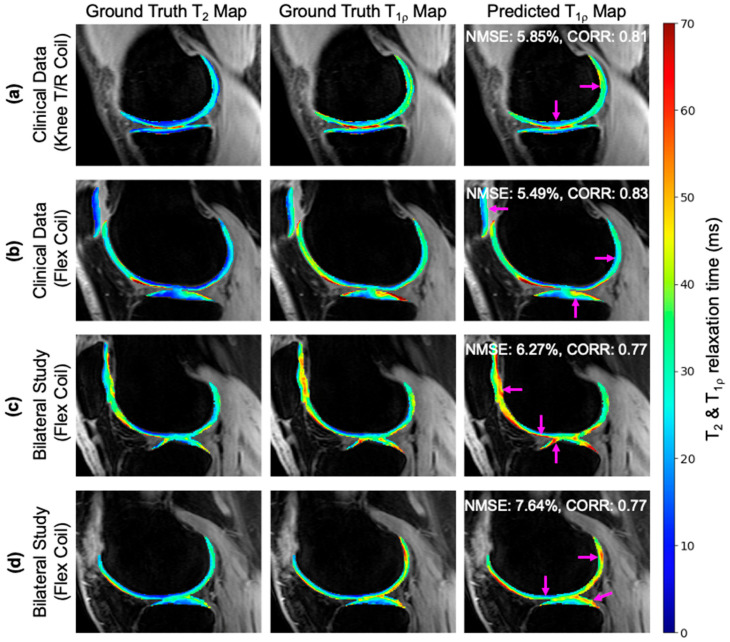
(**a**–**d**) Model inference was performed on data outside of the training data distribution to generate synthetic T_1p_ maps. Images are shown for 4 knees collected in (**a**,**b**) a clinical setting or (**c**,**d**) as part of a bilateral acquisition research study. Input ground truth T_2_ maps, ground truth T_1p_ maps, and predicted T_1p_ maps demonstrate the network effectively retained the elevation and textural patterns even though NMSE was higher than the development dataset. Regions marked by arrows showcase the network’s ability to synthesize T_1p_ maps despite varied relative differences in T_2_ map elevation.

Synthesis of new information was assessed in T_1p_ maps of four knees: two from the clinical dataset and two from the bilateral knee study (Figure 5). (a) For a clinical knee acquired using a knee T/R coil, the network used T_2_ maps to infer the appropriate intensity gradient of T_1p_ in the anterior horn and posterior horn relative to the central femoral cartilage. (b) For a clinical knee acquired with a flex coil, patterns were generally well maintained but the extent of T_1p_ elevation in the anterior femoral cartilage was not fully realized. (c,d) For bilateral study knees acquired with two flex coil arrays, T_1p_ map intensities are well synthesized, which is demonstrated (c) in the tibia and patellar relative to the central femoral cartilage, and (d) in the posterior femoral cartilage relative to the central cartilage. These four example cases have cartilage slice NMSE ranging from 5.85 to 7.64%, which was higher than the development dataset; yet, in all cases, the relative intensity patterns were still preserved.

#### 3.2.2. Image Generation Performance Evaluation

Similarity metrics reported in Table 3 exhibited a slight decrease in performance for out-of-distribution datasets (cohorts C and D) compared to held-out test data from the development dataset (cohorts A and B). For the entire clinical dataset acquired with a unilateral knee coil, performance metrics were better than the bilateral dataset. NMSE in cartilage tissue increased by 2.02% in comparison to the development dataset, and performance metrics were best for data collected with the same coil as the training dataset. For the bilateral dataset, NMSE in cartilage tissue increased by 4.85% compared to the development dataset. Consistent with trends in the development cohort, cartilage NMSE is lower than the NMSE across the entire knee volume.

#### 3.2.3. Quantitative Correlation Analysis

**Figure 6:**
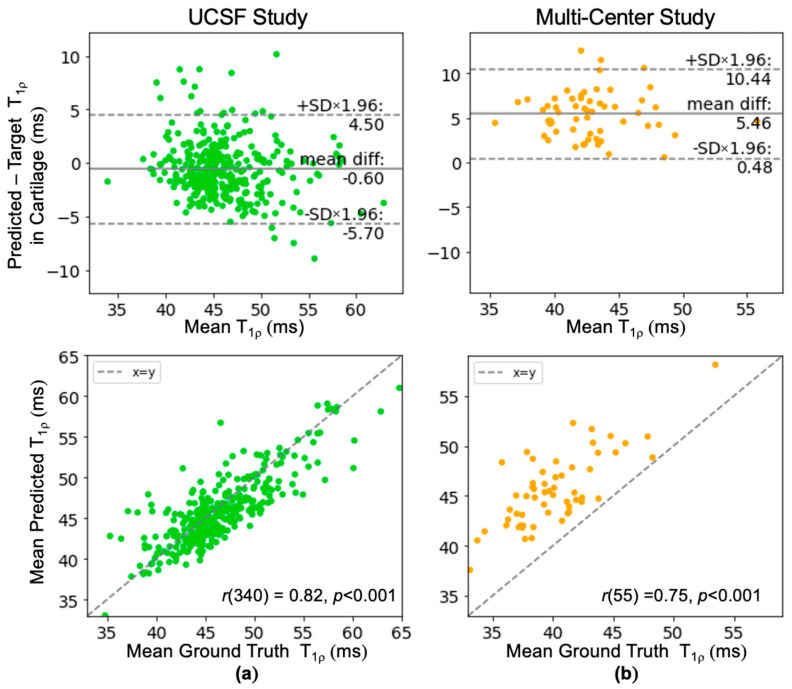
Bland-Altman and correlation plots of out-of-distribution data to evaluate the generalizability of the network. (**a**) Data acquired for the bilateral knee research study at UCSF using two knee coils simultaneously whereas training data was acquired with a unilateral coil. (**b**) Data acquired in a clinical setting had much greater variability in scanners and knee coils used.

Bland-Altman plots for out-of-distribution data show varied bias, wider limits of agreement (±4.98 ms or ±5.1 ms), and weaker correlation compared to in-distribution data, as expected (Figure 6). For data collected in a clinical setting (cohort C), there was minimal negative bias and ground truth T_1p_ values averaged 46.38 ± 4.46 ms, similar to the development dataset. For bilateral knee data (cohort D), the ground truth T_1p_ values averaged 40.13 ± 3.63 ms and the predicted T_1p_ values were on average 5.46 ms higher than the ground truth.

**Figure 7:**
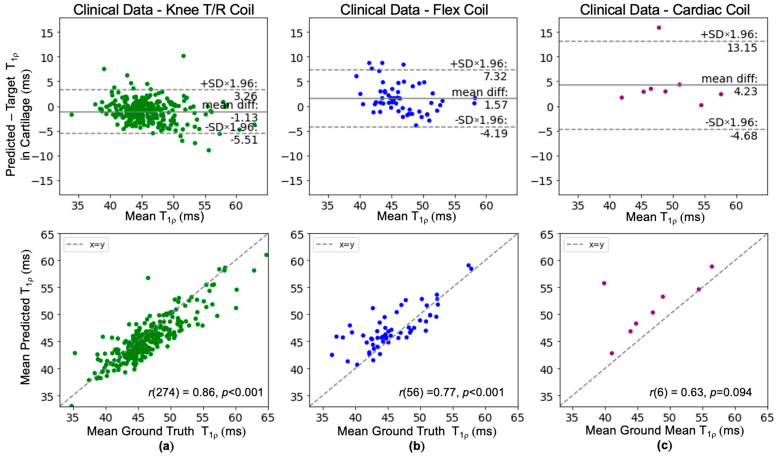
(**a**–**c**) Bland–Altman and correlation plots for T_1p_ images acquired using three different types of knee coils from the clinical dataset (cohort C). Given the knee T/R coil was used in the development dataset, the limits of agreement for the knee T/R coil were narrowest, larger for the flex coil, and widest for the cardiac coil.

Bland-Altman and correlation plots were also created to isolate the effect of the knee T/R, flex, and cardiac coil array on performance (Figure 7). A similar magnitude of bias was observed for the knee T/R coil (−1.13 ms) and flex coil (1.57 ms) while the cardiac coil had the largest bias (4.23 ms). The limits of agreement were slightly higher than training cohort limits for the knee T/R coil ±4.39 ms, higher for the flex coil ±5.75 ms, and even higher for the cardiac coil ±8.92 ms.

## 4. Discussion

This study presented one of the first networks for quantitative image synthesis in the musculoskeletal domain and conducted comprehensive performance evaluation across four cohorts, two of which had a slight variation in image acquisition settings, scanner, and coil arrays under unforeseen clinical and research settings. Despite these differences, the network generated synthetic T_1p_ maps well, as indicated by low NMSE and similar textures compared to ground truth maps for both healthy and OA knees. This work aimed to explore the performance of the development cohort held-out testing data as well as isolate the network’s tolerance to different inputs. Performance was measured in terms of local image intensities and global similarity metrics. Such analysis may capture both the benefits of synthesis as well as challenges with generalizability.

### 4.1. T_1p_ Synthesis Model Strengths

While there is some degree of correlation between T_2_ and T_1p_ relaxation times, prior work has demonstrated the value of utilizing both maps to probe cartilage morphology, particularly at the early stages of disease [23,56]. It has also been shown that T_1p_ is slightly more sensitive to mild OA than T_2_ [14]. Consequently, regions of cartilage with variation in texture and elevation patterns are both clinically interesting and challenging areas for synthesis.

Across all cohorts, the network proposed in this study was able to synthesize new information from T_2_ maps in areas exhibiting distinct T_1p_ intensity patterns relative to T_2_ (Figure 2 and Figure 5). Excellent inference with minimal NMSE in cartilage tissue was observed for in-distribution data while trends in T_1p_ texture and elevation changes relative to T_2_ were captured with slightly less accuracy for different scanners and coils. This suggests the network is least sensitive to research and clinical environments but does exhibit some sensitivity to scan equipment configurations. Proper reconstruction enables texture analysis of T_1p_ profiles that have the potential to detect early or local abnormalities indicative of OA that would otherwise go undetected based on morphological changes [57].

For the held-out test dataset, similarity metric performances indicated exceptional synthesis in relation to several benchmarks for scan/re-scan reproducibility, scan acceleration, and clinical significance. For the same MR system, T_1p_ map cartilage synthesis is limited by in vivo scan/re-scan reproducibility found to be 3.1% (range 1.0–1.7 ms RMS) across 3 sites and 16 knees for cartilage compartments [58]. Cartilage tissue NMSE was within the bounds of variability for re-scanning and the 5.7% quantification error rate was within the limits of clinical significance. Recall that cartilage defects such as lesions or meniscal tears are observed to elevate T_1p_ values within the entire cartilage compartment and surrounding areas [59]. Prior work has identified that 6.4% changes in cartilage T_1p_ [23,24] and 4% to 15% changes in cartilage compartments [27] can be clinically significant for OA diagnosis and management.

Moreover, synthesis performance is comparable to image reconstruction with an acceleration factor of two, as the generated maps are obtained with roughly half the echo images, which is analogous to reducing scan time by half. Reduction in scan time decreases acquisition cost, making clinical adoption of T_1p_ maps more feasible. Prior work has shown that further acceleration of the knee MAPSS sequence by a factor of two introduced 1.49 ms bias and confidence intervals of ±4.55 ms in the cartilage region [60]. Bland–Altman plots revealed that the network in this study achieved minimal bias (range 0.10–0.45 ms) an order of magnitude lower than similar maps reconstructed with R = 2 acceleration. Although the limits of agreement were wider than the reported range of scan/re-scan variability for fully acquired T_1p_ maps, they were tighter than the limits reported for R = 2 reconstructions of T_2_ maps. These results demonstrate the feasibility of synthesis for T_1p_ maps and indicate that synthetic images outperform several reproducibility benchmarks. Additionally, compartment-wide analysis using synthetic images holds clinical value.

For all cohorts, similarity metric performances were highest for cartilage-specific analysis compared to whole image volume assessment likely due to the weighted loss function and noise in the image background. Bias for T_1p_ estimation in these compartments remained minimal while the limits of agreement widened. This may occur due to fewer voxels contributing to the compartment average instead of the entire cartilage region, thereby increasing Bland-Altman sensitivity to variability but not changing the bias. Despite smaller cartilage compartment regions, the network demonstrated robust synthesis since the performance was consistent across mean T_1p_ values for both UCSF (cohort A) and the muti-center study (cohort B) which had a more varied distribution of T_1p_ values in part due to segmentation quality.

### 4.2. Synthesis Generalizability Assessment on External Datasets

The proposed study has established an initial working baseline for T_1p_ map synthesis. However, widespread usage is limited by the network’s ability to generalize to datasets with varied, previously unseen acquisition settings and environments. To investigate network generalizability, synthetic T_1p_ maps were generated from data collected in a clinical setting in addition to standard-of-care imaging and from data collected in a research setting using two flex coils simultaneously for bilateral knee acquisition. The loss function in the proposed network was optimized using both T_1p_ and T_2_ map values, which are prone to variance dependent upon acquisition parameters. More specifically, scanner and coil hardware systems may cause slight differences in B0/B1 inhomogeneity patterns that change proton resonance frequencies and excitation profiles. As a result, effects on T_1p_/T_2_ preparations can be different [61,62] such that T_1p_/T_2_ signal decay is affected disproportionately. In addition, multi-coil combination methods for bilateral MRI acquisition were different from others (adaptive coil combination versus standard sum-of-square combination). These reconstruction method variations might also result in a bias for synthetic T1rho maps from bilateral acquisitions. Model performance decreased in both settings but more so in the bilateral study setting likely due to these differences. Nevertheless, these variations provide valuable insight into the synthesis model.

Data from the clinical setting (cohort C) exhibited similarities to the training dataset with regard to patient population demographics, unilateral knee acquisition, type of scanner used, and type of knee coil used (81% cohort C, n = 278). Differences arose when data were collected using various receive coils: 17% flex coil (n = 57) and 2% cardiac coil to accommodate patient geometries (n = 9). Overall, the network maintained minimal bias (−0.6 ms) for clinical data which was most like the multi-center study bias (−0.45 ms) whose data were primarily acquired on the same scanner.

For clinical data stratified by coil array, network performance was best for data acquired with the same knee T/R coil as the training data, slightly declined for the flex coil, and was the poorest for the cardiac coil. Data acquired with the same coil had bias (−1.13 ms) within the range of scan/re-scan reproducibility. The effect of MR scanner and coil on map values has been quantified by Li et al. in a reproducibility study. The study found in vivo T_1p_ and T_2_ values for healthy subjects did not have significant differences across sites but did vary depending on the MR system (difference of 2.8 ms for T_1p_ and 2.9 ms for T_2_ between HDx long bore and MR750 wide bore scanners) and knee coil (difference of 2.8 ms for T_1p_ and 4 ms for T_2_ between 16PAR flex and QT8PAR knee coils) [38]. For the flex coil data, bias was within the range of scan–re-scan reproducibility. However, for the limited cardiac coil, the bias was greater potentially due to significant observable differences in image SNR and larger patient body shape effect on magnetic field inhomogeneities. Performance changes in limits of agreement and NMSE may be explained by coil differences, such as the use of differing transmit systems (knee coil versus body coil excitation), suggesting future synthesis algorithms may benefit from incorporating scanner information into the network. However, for this network quantification, error rates increased beyond the limit for clinical significance (clinical—all: 11.0%; clinical—knee T/R coil: 9.5%; clinical—flex coil: 12.4%; clinical—cardiac coil: 19.2%) warranting further exploration prior to quantitative evaluation yet qualitative assessment remains feasible.

Primary differences between the bilateral acquisition dataset and training dataset included simultaneous acquisition from two flex coils, updated coil combination software on the scanner, and an older patient population averaging 18 years senior without ACL tear or reconstruction. Prior work by Verschueren et al. found significant increases in T_2_ relaxation times with both age and BMI across 109 patients [63], making them covariates of quantitative T_2_ mapping for OA detection. While performance was expected to match that of the clinical dataset acquired with the flex coil, performance decreased by +1.85% cartilage NMSE and +3.89 ms bias. At this time, reproducibility metrics involving bilateral acquisition of knee MRI using flex coils are not available. Nevertheless, the differences in the study suggest future work will benefit from a reproducibility study that investigates potential increases in B0/B1 inhomogeneities over two knee volumes and the effects of coil combination software methods. Additionally, age-related differences or usage of two flex coils may have contributed to the overestimation of predicted T_1p_ maps. This error can hypothetically be overcome with inference testing of an age-matched population, which was not performed in the proposed study due to the unavailability of such data.

### 4.3. Network Limitations

In this study, the network performance was constrained by variability in the training dataset, which is consistent with the limitations seen in algorithms trained on local datasets [64]. Despite challenges in obtaining diverse datasets, future endeavors aiming to create clinically useful and broadly applicable networks should prioritize training on datasets containing greater diversity in MR hardware and image reconstruction software. While comparing state-of-the-art algorithms is common practice, this work demonstrates the value of assessing performance on external, inference-only datasets to develop models with greater utility. Additionally, stringent cartilage segmentations were not required for synthesis evaluation. As a result, this study did not address clinically significant quantitative values although such analyses may be enabled by synthesis in future work.

### 4.4. Future Direction

Future work may also benefit from the consideration of alternative preprocessing techniques and network architectures that have the potential to be more robust to scanner and coil differences. While 3D V-Nets require additional computational recourses, inputting 3D images as opposed to 2D may allow the network to infer systematic changes in B0/B1 inhomogeneities and the effect of metal artifacts. An end-to-end approach has improved network task performance in other studies and may be explored by synthesizing T_1p_ and T_2_ maps directly from echo images [65]. Alternatively, model pre-training on a subset of study-specific data or model fine-tuning may improve generalizability across MR scanners, knee coils, patient cohorts, and magnet field strength [66].

Modification to network architectures may include the exploration of variational U-Nets [46,67,68] generative adversarial networks (GANs) [69,70], variational autoencoders [71,72], transformer-based models [73], and other state-of-the-art methods. Additional network modifications may include the incorporation of a contrastive loss term [74] and data augmentation techniques [75]. While this study opted for a data-driven approach to image synthesis, further optimization could be achieved by the incorporation of a contrastive loss term that leverages explicit knowledge of acquisition parameter details (scanner, coil array, repetition times, echo times, etc.) and image SNR. The loss can be integrated into a network discriminator encouraging the generator to produce a scanner-agnostic image, or between the network encoder and decoder, prompting the network to extract features that are agnostic to the scan system.

Prior to widespread adoption, further network development to achieve quantification error rates within the range of clinically relevant changes could provide more confidence in synthetic imaging. Additionally, advancements in standardized coil arrays or calibration could promote greater consistency in T_1p_ and T_2_ relaxation times, necessary for OA biomarker validation. This need is consistent with findings from a meta-analysis across 55 studies [76]. Such standardization would reduce non-physiological variability thereby presenting a simpler mapping problem for image synthesis.

### 4.5. Future Application towards Clinical Biomarker Extraction

Alternatively, deep learning algorithms offer high reproducibility and may pose as an alternative for quantitative imaging biomarker standardization and faster clinical translation [77]. Due to the heterogeneity of knee OA, stratification of patient subpopulations based on OA disease onset, stage, and risk of progression is a critical next step to improve early detection and care [78]. By probing tissue cellularity and molecular composition, T_1p_ maps have the potential to define diagnostic criteria for OA as well as outcome measures.

However, the Quantitative Imaging Biomarkers Alliance observed limited translation of early-stage knee OA biomarkers into clinical practice due to “variability across devices, sites, patients and time” and are spearheading standardization efforts [30]. Previous studies on MR fingerprinting and quantitative susceptibility mapping have used deep learning algorithms to infer tissue properties from MR signals with the potential for improved accuracy and consistency [44,77,79]. Image synthesis can aid efforts to standardize T_1p_ and T_2_ measurements of knee cartilage by capturing complex non-linearities between the two sequences to make T_1p_ maps accessible. The U-Net proposed in this work, and CNNs in general, create image feature representations highly attuned to the input images. Once a standardized T_2_ map is established, quantitative T_1p_ biomarkers generated by a CNN can quickly be extracted for data-driven validation and integrated into clinical workflows as a clinical decision-making tool with minimal barriers associated with multi-site data harmonization [78] and clinical translation [80].

## 5. Conclusions

The network was able to generate synthetic T_1p_ images from T_2_ images with excellent fidelity to ground truth T_1p_ images. For data collected across multiple institutions and studies, textures were preserved and the limits of agreement for T_1p_ NMSE were below the limits of clinical relevance. The generalizability of the network showed decreased performance for data acquired in less controlled external datasets, yet variation between MR scanners and coils may account for these differences.

This work shows the capability of deep learning to extract additional diagnostic information from already acquired T_2_ maps. With further development, a pipeline like this creates new possibilities for population studies like the OA Initiative, which can add to the characterization of OA, potentially facilitate clinical translation, and complement efforts to establish quantitative imaging biomarkers. Additionally, this study shows the promise of deep learning in accelerating imaging protocols through domain adaptation as opposed to more common reconstruction, standardization, and calibration approaches.

## Figures and Tables

**Table 1:** Cohort demographics and scan equipment breakdown for 897 knees in this study. Age and BMI (mean ± stdev.) are known covariates of OA indication from relaxation times.

Cohort	A	B	C	D
**Study**				
Institution	UCSF	UCSF, HSS, Mayo	UCSF	UCSF
Acquisition setting	Research	Research	Clinical	Research
**Demographics**				
Number of knees	273	235	343	46
Number of patients	75	175	321	23
Age	30 ± 8	29 ± 14	37 ± 13	58 ± 13
Males/Females	10:7	10:8	7:5	10:9
BMI	72.9 ± 13.1	74.3 ± 12.1	77.3 ± 15.9	74.2 ± 16.1
**Scanners and Coil Combination Method**				
GE Signa MR750 (GE Healthcare, Waukesha, WI, USA), sum-of-square coil combination	*n* = 56	*n* = 155	*n* = 343	---
GE Signa MR750W (GE Healthcare, Waukesha, WI), sum-of-square coil combination	*n* = 216	*n* = 67	---	---
GE Signa PET-MRI (GE Healthcare, Waukesha, WI), sum-of-square coil combination	---	*n* = 13	---	---
GE Signa Premier (GE Healthcare, Waukesha, WI), adaptive coil combination	---	---	---	*n* = 46
**Knee Coil**				
8-channel transmit/receive knee coil array (In-Vivo Corp., Gainesville, FL, USA)	*n* = 273	*n* = 235	*n* = 278	---
16-channel medium GEM flex-coil array (Neo-Coil, Pewaukee, WI, USA)	---	---	*n* = 32	*n* = 46 (2 simultaneous acquisitions)
16-channel large GEM flex-coil array (Neo-Coil, Pewaukee, WI)	---	---	*n* = 25	---
8-channel cardiac coil array (GE Healthcare, Waukesha, WI)	---	---	*n* = 8	---

**Table 2:** Similarity metrics between ground truth and predicted T_1p_ maps for patients who participated in the UCSF study, multi-center study, and across both studies.

Similarity Metric	NMSE (%)		PSNR	CORR	SSIM
*avg ± stdev. across patients*	*volume*	*cartilage*	*cartilage*	*cartilage*	*volume*
All Studies	4.18 ± 1.97	2.41 ± 1.51	23.99 ± 1.98	0.87 ± 0.08	0.62 ± 0.06
UCSF Study	4.84 ± 2.42	2.61 ± 1.47	24.00 ± 1.78	0.85 ± 0.07	0.62 ± 0.06
Multi-center Study	3.42 ± 0.67	2.18 ± 1.53	23.97 ± 2.17	0.88 ± 0.08	0.62 ± 0.05

**Table 3:** Synthetic T_1p_ maps were generated for out-of-distribution data to test model generalizability. Performance was assessed per knee coil used during image acquisition. Across all similarity metrics, performance decreased slightly compared to the development test set. Similarity metrics were best for data collected with the same knee T/R coil as the training dataset.

Similarity Metric	*NMSE (%)*		*PSNR*	*CORR*	*SSIM*
*avg ± stdev. across patients*	*volume*	*cartilage*	*cartilage*	*cartilage*	*volume*
Clinical Data—All	7.35 ± 3.74	4.43 ± 3.61	20.63 ± 2.46	0.84 ± 0.11	0.54 ± 0.08
Clinical—Knee T/R Coil	7.93 ± 3.85	4.17 ± 3.2	20.87 ± 2.37	0.85 ± 0.10	0.57 ± 0.06
Clinical—Flex Coil	4.57 ± 1.28	5.41 ± 4.99	19.89 ± 3.00	0.79 ± 0.17	0.42 ± 0.05
Clinical—Cardiac Coil	7.12 ± 2.96	6.26 ± 3.48	17.93 ± 2.37	0.79 ± 0.10	0.51 ± 0.08
Bilateral Study—Flex Coil	8.93 ± 3.17	7.26 ± 3.55	18.20 ± 2.31	0.76 ± 0.14	0.53 ± 0.06

## Data Availability

Code for this project is available at https://github.com/michelle-tong18/synthetic-t1rho-maps n. Dataset access is available from the coauthors upon reasonable request.

## Appendix A

**Table A1:** Similarity metrics between predicted and ground truth T_1p_ maps for UCSF (cohort A) and multi-center (cohort B) data. Metrics were calculated from the average T_1p_ in entire cartilage region and six cartilage compartments: medial femoral, lateral femoral, medial tibial, lateral tibial, trochlea, and patellar cartilage. Between the entire cartilage region and compartments, performance was quite similar apart from a slight decline in similarity for the multi-center trochlea and patellar cartilage.

Metric	*NMSE (%)*		*PSNR*		*CORR*	
*avg ± stdev.*	*UCSF*	*Multi-Center*	*UCSF*	*Multi-Center*	*UCSF*	*Multi-Center*
Cartilage	2.61 ± 1.47	2.18 ± 1.53	24.00 ± 1.78	23.97 ± 2.17	0.85 ± 0.07	0.88 ± 0.08
MF	2.68 ± 1.86	3.08 ± 2.40	22.78 ± 2.35	18.94 ± 2.17	0.82 ± 0.11	0.91 ± 0.07
LF	2.74 ± 1.72	2.86 ± 1.74	23.07 ± 2.16	19.21 ± 2.03	0.84 ± 0.08	0.91 ± 0.05
MT	4.24 ± 3.77	3.02 ± 2.70	22.13 ± 2.96	18.99 ± 2.76	0.82 ± 0.17	0.89 ± 0.07
LT	3.52 ± 3.08	2.71 ± 1.91	23.02 ± 2.49	19.05 ± 2.64	0.87 ± 0.13	0.85 ± 0.19
TROC	2.06 ± 1.03	4.78 ± 2.65	24.18 ± 1.99	16.77 ± 2.31	0.87 ± 0.07	0.81 ± 0.11
PAT	2.04 ± 1.32	5.79 ± 4.39	24.42 ± 2.47	16.83 ± 2.4	0.88 ± 0.06	0.83 ± 0.12
